# Potential serum biomarkers for glioblastoma diagnostic assessed by proteomic approaches

**DOI:** 10.1186/s12953-014-0047-0

**Published:** 2014-09-24

**Authors:** Ionela Daniela Popescu, Elena Codrici, Lucian Albulescu, Simona Mihai, Ana-Maria Enciu, Radu Albulescu, Cristiana Pistol Tanase

**Affiliations:** Biochemistry-Proteomics Department, Victor Babes National Institute of Pathology, no 99-101 Splaiul Independentei, 050096 Sector 5, Bucharest, Romania; Department of Biochemistry and Molecular Biology, Faculty of Biology, University of Bucharest, no. 91-95 Splaiul Independentei, 050095 Sector 5, Bucharest, Romania; Cellular and Molecular Medicine Department, Carol Davila University of Medicine and Pharmacy, no 8 B-dul Eroilor Sanitari, 050474 Sector 5, Bucharest, Romania; National Institute for Chemical Pharmaceutical R&D, 112 Calea Vitan, 031299 Sector 3, Bucharest, Romania; Current address: Virology Division, Department of Infectious Diseases and Immunology, Faculty of Veterinary Medicine, Utrecht University, Utrecht, The Netherlands

**Keywords:** SELDI-ToF MS, LC-MS/MS, Glioblastoma, Biomarkers, S100A8, S100A9, CXCL4

## Abstract

**Background:**

The rapid progress of proteomics over the past years has allowed the discovery of a large number of potential biomarker candidates to improve early tumor diagnosis and therapeutic response, thus being further integrated into clinical environment. High grade gliomas represent one of the most aggressive and treatment-resistant types of human brain cancer, with approximately 9–12 months median survival rate for patients with grade IV glioma (glioblastoma). Using state-of-the-art proteomics technologies, we have investigated the proteome profile for glioblastoma patients in order to identify a novel protein biomarker panel that could discriminate glioblastoma patients from controls and increase diagnostic accuracy.

**Results:**

In this study, SELDI-ToF MS technology was used to screen potential protein patterns in glioblastoma patients serum; furthermore, LC-MS/MS technology was applied to identify the candidate biomarkers peaks. Through these proteomic approaches, three proteins S100A8, S100A9 and CXCL4 were selected as putative biomarkers and confirmed by ELISA. Next step was to validate the above mentioned molecules as biomarkers through identification of protein expression by Western blot in tumoral *versus* peritumoral tissue.

**Conclusions:**

Proteomic technologies have been used to investigate the protein profile of glioblastoma patients and established several potential diagnostic biomarkers. While it is unlikely for a single biomarker to be highly effective for glioblastoma diagnostic, our data proposed an alternative and efficient approach by using a novel combination of multiple biomarkers.

## Background

The rapid progress of proteomics over the past ten years has allowed the discovery of a vast number of potential biomarker candidates; however, the majority of novel candidates has not been integrated yet into clinical environment [1].

Glioblastoma is the most common primary brain tumor associated with a relatively short survival rate; the median rate of survival for glioblastoma multiforme patients is only 9 to 12 months [2]. In spite of the great challenge represented by early detection of asymptomatic glioblastoma through high cost diagnostic imaging methods, the development of a convenient, sensitive, and cost-effective diagnostic strategy is necessary [3].

Glioblastomas represent more than 40% of all primary central nervous system neoplasms. Although all glioblastomas derive from glial precursors, they vary considerably in morphology, location, genetic alterations, and response to therapy [4,5]. Histopathology exams are the gold standard for the typing and grading of glioblastomas, however this histological classification remains unsatisfactory because of low reproducibility and poor precision in terms of prognosis, as evidenced by large inter-observer variability [6].

The identification of biomarkers for early tumor growth, recurrence, and therapeutic response are of great interest in oncology. Considerable efforts are currently focused on methods for early tumor detection, including those involving detection of specific proteins or proteomic profiles from biopsies and especially from serum/plasma [7,8].

Surface-enhanced laser desorption/ionization time-of-flight mass spectrometry (SELDI-ToF MS) method is currently being developed to meet the demand for a higher throughput in clinical settings. Numerous studies have already provided evidence that this methodology can be used to uncover proteomic expression patterns linked to a disease state [9]. This platform has been successfully applied for the identification of serum biomarkers in different cancer types and has recently shown high promise in the detection of early-stage cancers [10].

Recent studies have shown that cytokines and chemokines are produced in the tumour microenvironment and have highlighted their key role played in cancer pathogenesis. Cytokine and chemokine panels might be used as a prognostic marker for various cancers, with abnormally modified levels associated with a poor prognosis and potential tumor metastasis or recurrence [11]. There are many studies in this regard that provide valuable information about the role of these biomarkers in different types of malignancies and their potential benefit as an adjuvant therapy [12,13].

**Figure 1:**
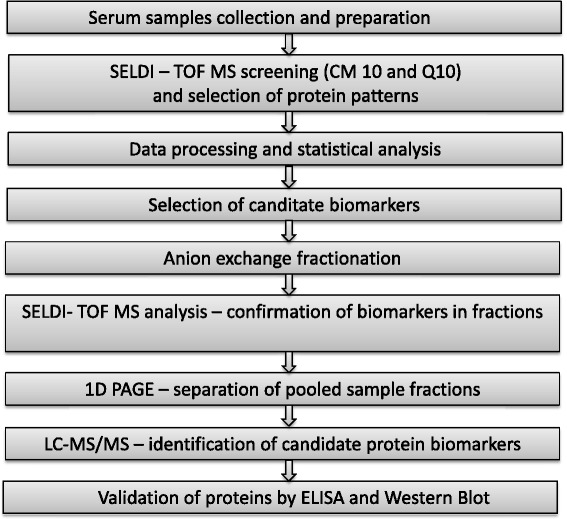
**The work-flow followed in biomarker discovery.**

In view of this, our study used a comparative proteomic analysis based on SELDI-ToF MS, LC-MS/MS and Western blot in order to obtain protein profiles and identify potential biomarkers from serum of glioblastoma patients and controls (Figure 1).

## Results

### Identification of differentially expressed proteins based on SELDI-ToF MS

In our study SELDI-ToF MS technology was used to screen potential protein patterns in glioblastoma patients serum, furthermore, LC-MS/MS technology was applied to identify the candidate biomarkers peaks. After the multiple set of proteomic approaches was made, the validation of the confirmed biomarkers was performed by enzyme-linked immunosorbent assay (ELISA) and Western blot.

**Figure 2:**
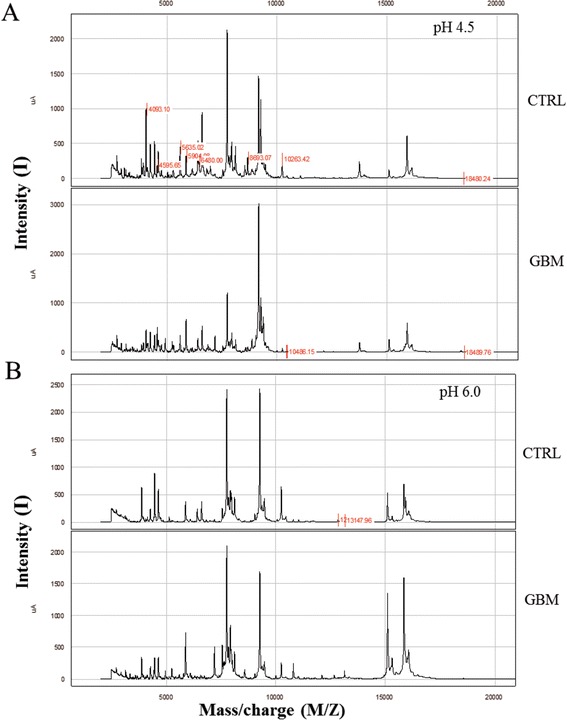
**Selection of SELDI – ToF spectra according to pH parameter.** Exemples of SELDI-ToF mass spectra obtained under weak cationic exchange (CM10) arrays, pH 4.5 **(A)** and pH 6.0 **(B)** of pooled serum (n = 5) from controls and patients with glioblastoma.

Spectra were selected on the basis of the largest number of peaks present at each pH (3.5-7.0 for CM10 and 4.5-8.0 for Q10) and in terms of each peak’s relative intensity. The goal in the beginning was to select a small number of pH buffer conditions to be used to analyze all samples. CM10 at pH 4.5 and 6.0 were selected for individual serum sample analysis (Figure 2).

Spectral data were analysed using ProteinChip Data Manager v. 3.0.7 Software to generate peak mass clusters and for delineation of candidate biomarker peaks.

The data were first analysed by a univariate analysis tool, which clusters the peaks and determines the *p values* for each array condition. After exclusion of peaks with low signal-to-noise ratio a number of 73 protein clusters (range 2–55 KDa) have been identified; 6 potentially relevant clusters were selected by further analysis on CM10, pH 4.5 (p values < 0.05). The molecular weights (MW) of the identified clusters were: **8143.15; 2948.04; 23466.27; 6440.01; 3092.01; 9192.84** - CM10 for pH 4.5. Applying the same conditions on CM10, pH 6.0, a number of 79 protein clusters was found (range 2–33 KDa), out of which 5 clusters were selected: **3892.55; 10836.09; 13153.66; 15868.12; 28114.62** (Table 1).

**Table 1:** **Differentially expressed protein peaks in glioblastoma as compared to control**

**No.**	**Mass (m/z)**	**p-value**	**ROC area**	**Expression change in glioblastoma**
1	8143.15	<0.001	0.747619	↓
2	2948.04	<0.005	0.673333	↓
3	23466.27	<0.05	0.648571	↑
4	6440.01	<0.05	0.648571	↓
5	9192.84	<0.001	0.859048	↑
6	3092.01	<0.001	0.859048	↓
7	3892.55	<0.001	0.908571	↓
8	10836.09	<0.001	0.811619	↑
9	13153.66	<0.001	0.911429	↑
10	15868.12	0.001	0.829524	↑
11	28114.62	<0.001	0.809524	↑

m/z: mass/charge ratio of the protein peak; ↑: protein level was increased in glioblastoma as compared to control; ↓: protein level was decreased in glioblastoma as compared to control.

### Anion exchange fractionation

Serum pools were fractionated using anion exchange columns and the protein profile from different fractions eluates (F1-F6) was analysed by SELDI-ToF-MS. This sequence ensures a great opportunity to obtain simplified proteomes that includ one or more biomarker peaks.

**Figure 3:**
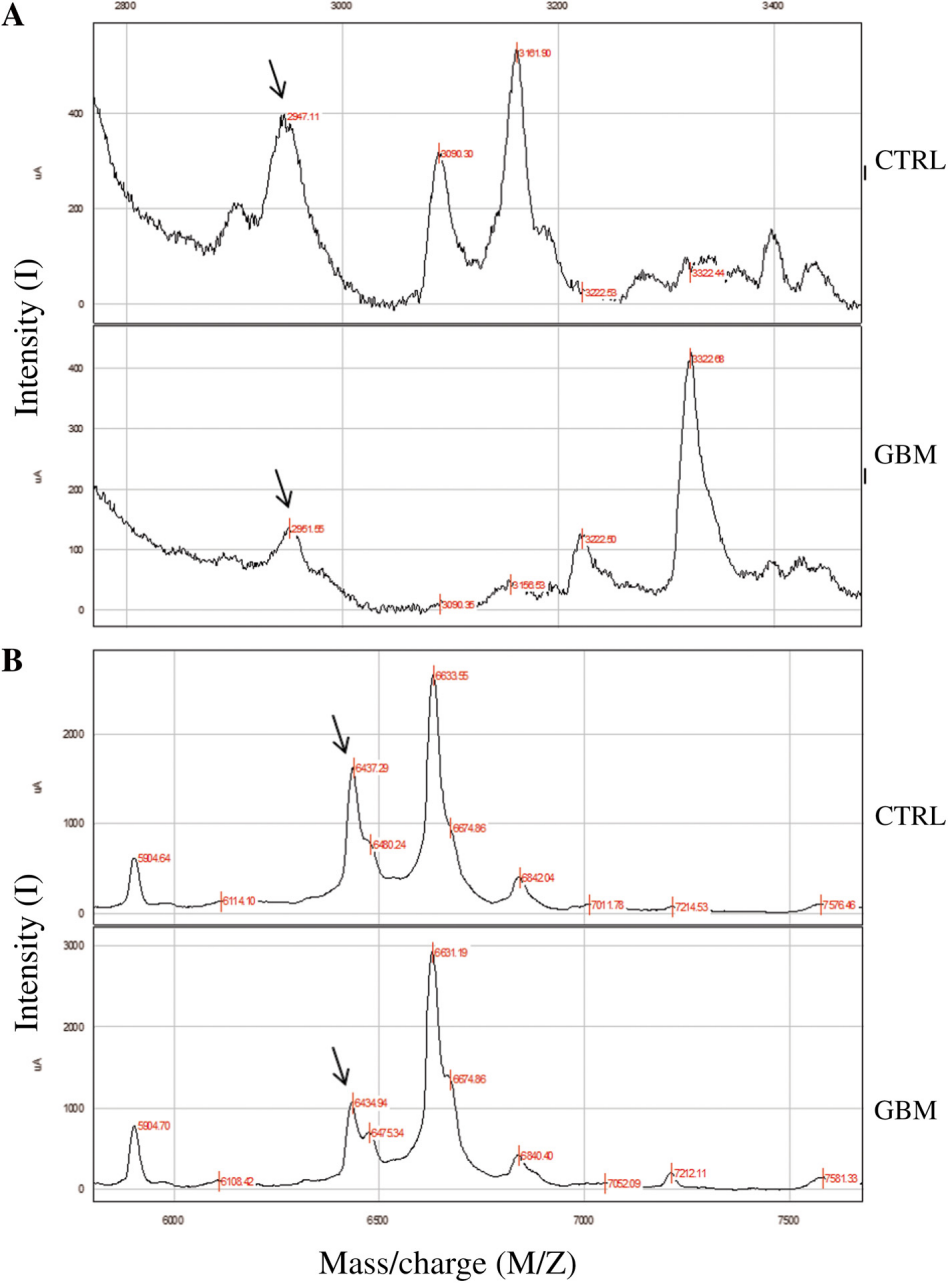
**Reproducibility of SELDI – ToF mass spectra.** Different relative intensities of peptide peaks between glioblastoma diagnosed (n = 35) and control group (n = 30); **(A)** down-regulated peptide with m/z 2948.04 in glioblastoma patients. **(B)** down-regulated peptide with and 6440.01 in glioblastoma patients.

**Figure 4:**
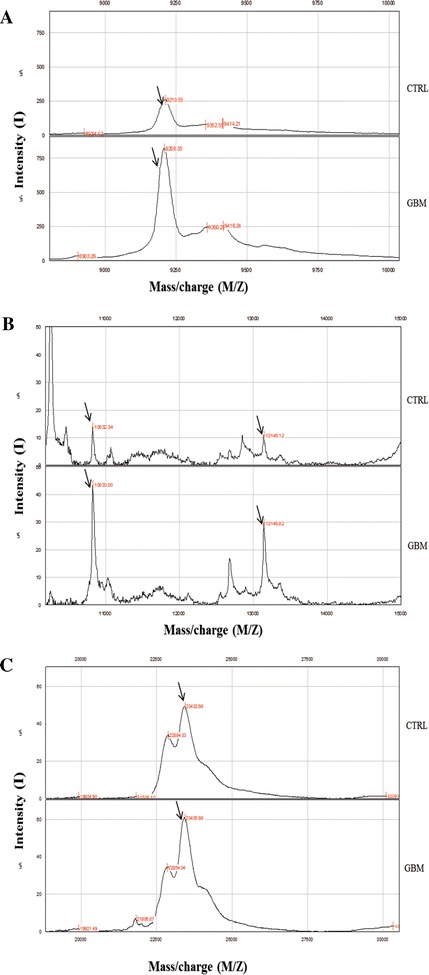
**Reproducibility of SELDI – ToF mass spectra.** Different relative intensities of peaks in glioblastoma diagnosed (n = 35) *vs* control group (30); **(A)** peaks at m/z 9192.84 were up-regulated in glioblastoma patients. **(B)** peaks at m/z 10836.09, 13153.66 were up-regulated in glioblastoma patients. **(C)** peaks at m/z 23466.27 were up-regulated in glioblastoma patients.

Our results showed that one group of peptide with m/z 2948.04 and 6440.01 were down-regulated in glioblastoma patients *vs* control (Figure 3) and another group with m/z 9192.84, 10836.09, 13153.66 and 23466.27 were up-regulated in glioblastoma patients *vs* control (Figure 4). These two groups were futher analyzed on 1D-PAGE.

### Identification of biomarkers

The next step was to identify the most abundant proteins in 1D PAGE and then work backwards to find their mass matches in the SELDI spectra.

The intensity of the bands in 1D PAGE (excluding 55 and 26 kDa) was not very strong especially in the lower mass range where the majority of the SELDI biomarkers are located. In addition, calibration of mass based on the position of mass markers was performed. In order to delineate potential gel bands corresponding to SELDI biomarker peak masses, next step was to align pseudo-gel image representations of the SELDI spectra containing biomarkers alongside the 1D PAGE images.

Given the fact that the results did not show a reliable one for one match, a more conservative approach was taken by cutting every visible gel band so that all bands in the <28 kDa region could be further identified by LC-MS/MS.

Using the assumption in this SELDI/gel based approach that gel bands correspond in actual mass (as opposed to mass indicated by migration) to SELDI peaks, the identification of each protein in the gel bands allows correlation with the database mass based on core amino acid composition. Following protein identification by LC-MS/MS, specific antibodies were used for the validation of candidate biomarkers.

### Data processing

#### LC-MS/MS

The data files (.raw) were converted into mascot generic files using the MassMatrix File Conversion Tool (Version 2.0; http://www.massmatrix.net) for input into the Mascot searching algorithm (Matrix Science). The data files were searched against both SwissProt (v. 2010_06) with human taxonomy using the following search criteria: tryptic peptides with up to one missed cleavage and carbamidomethylation of cysteines and oxidation of methionines, which were set as variable modifications.

We have compared the masses of the identified proteins by LC-MS/MS with masses of SELDI-ToF MS derived biomarkers. Given the mass tolerance of SELDI (0.05%), there are interesting mass similarities with SELDI markers at 10835/6 found in the databases mass of protein S100A8 and a cluster of markers around 13152 and 13769 with protein S100A9, CXCL4 and CXCL7. All these proteins have been implicated separately as biomarkers of cancer.

The table below contains the selected identities of proteins obtained according to our search criteria (Table 2).

**Table 2:** **Protein identification**

**Protein name**	**Protein description**	**Protein score**	**Protein mass**	**Peptide expressed m/z**	**Peptide sequence**
S100A8_HUMAN	Protein S100-A8 OS = Homo sapiens GN = S100A8 PE = 1 SV = 1	164	10828	637.6	ALNSIIDVYHK
		164	10828	711.64	LLETECPQYIR
		164	10828	1196.38	ELDINTDGAVNFQEFLILVIK
S100A9_HUMAN	Protein S100-A9 OS = Homo sapiens GN = S100A9 PE = 1 SV = 1	287	13234	486.45	LTWASHEK
		287	13234	728.82	LGHPDTLNQGEFK
		287	13234	808.63	QLSFEEFIMLMAR
		287	13234	872.03	VIEHIMEDLDTNADK
		287	13234	904.41	NIETIINTFHQYSVK
PLF4_HUMAN	Platelet factor 4 OS = Homo sapiens GN = PF4 PE = 1 SV = 2	170	10838	667.44	ICLDLQAPLYK
		170	10838	731.77	KICLDLQAPLYK
		170	10838	789.7	AGPHCPTAQLIATLK
CXCL7_HUMAN	Platelet basic protein OS = Homo sapiens GN = PPBP PE = 1 SV = 3	105	13885	863.03	GKEESLDSDLYAELR
		254	13885	529.22	ICLDPDAPR
		254	13885	551.13	NIQSLEVIGK
		254	13885	592.95	KICLDPDAPR
		254	13885	785.48	GTHCNQVEVIATLK
		254	13885	863.27	GKEESLDSDLYAELR

Proteins identified in 1D-PAGE bands, which correspond to the molecular weights of differentially expressed proteins in glioblastoma to controls found by SELDI-ToF MS analysis (database with the Mascot algorithm).

### Validation of proteins by ELISA

**Figure 5:**
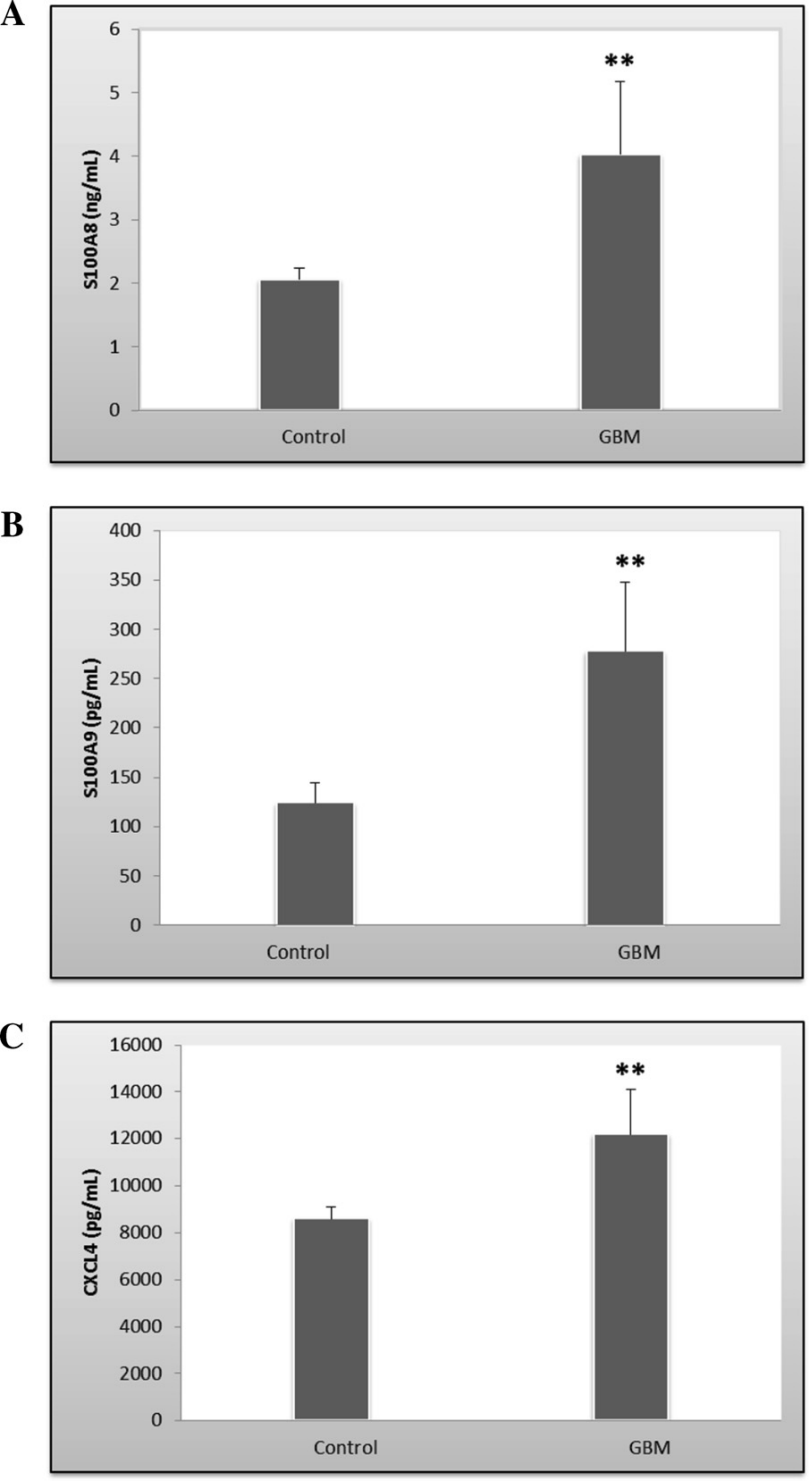
**Serum levels of S100A8 (A), S100A9 (B) and CXCL4 (C) as quantified by ELISA.** The bars represent the mean values calculated for the glioma group (n = 35) vs. controls (n = 30). Error bars represent +/− standard deviation. Statistical significance was set at **p < 0.01 (One-Way Anova).

Serum levels of S100A8, S100A9 and CXCL4 were validated using ELISA assay. Significantly increased levels of analyzed biomarkers were found in glioblastoma patients serum when compared to control group. For S100A8, the average value in glioblastoma patients was 4.03 ng/mL vs 2.06 ng/mL in controls (Figure 5A). S100A9 from glioblastoma subjects had an average value of 277.72 pg/mL, almost twice the average value of controls (124.25 pg/mL) (Figure 5B). Significant differences were also observed between serum levels of CXCL4 in patients with glioblastoma (12192.29 pg/mL) and controls (8608.24 pg/mL) (Figure 5C).

### Validation of proteins by Western blot

S100A9 and CXCL4 biomarkers were also validated using Western blot assay. As opposed to ELISA method, Western blot is less sensitive, needs a larger amount of protein and is semi-quantitative. Therefore, in order to save biological material, which is hard to obtain and difficult to preserve in optimal conditions that will not interfere with subsequent mass spectrometry analysis (such as addition of protease inhibitors or detergents), we chose one member of each family (S100 and chemokine, respectively) to be validated by blotting.

**Figure 6:**
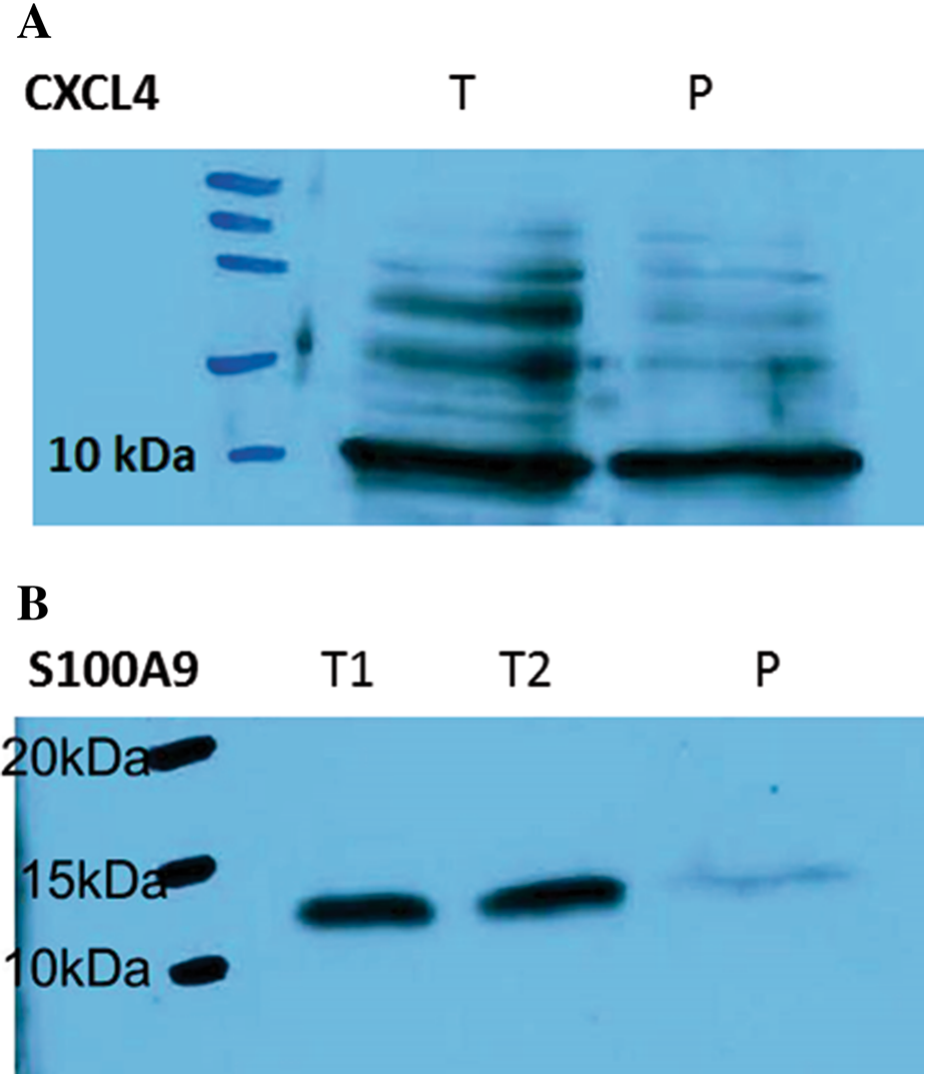
**Validation of proteins by Western blot.** CXCL4 **(A)** and S100-A9 **(B)** expression in tumoral (T) and peritumoral (P) tissues of glioblastoma.

As shown in Figure 6A, CXCL4/PF-4 was detected by Western blot in both tumoral and peritumoral tissue with increased expression in tumoral tissue.

At the same time, Western blot assay has confirmed that S100A9/Calgranulin B shown an increased expression in tumor tissues as opposed to peritumoral tissues (Figure 6B). We have analyzed two different tumor samples and reported herein an equally strong signal, in concordance with the low SD values found in ELISA analysis.

## Discussion

In recent years, the field of proteomics has been rapidly developing, due to the promising perspective of fast, multi-marker analysis, using small amounts of different biological samples. Clinical proteomics is primarily focused on the identification of possible biomarkers for diagnostic and/or disease progression from biological samples, such as body fluids (e.g. serum, cerebrospinal fluid), cells and tissues biopsies. Oncology is one of the main clinical branches for which discovery and development of biomarker panels, detected through simple, non-invasive or minimally invasive methods, is an important goal. Robust and efficient biomarker identification methods, a priority in clinical oncology research, can be implemented to identify cancer risk, improve early diagnostic and facilitate accurate grading and treatment monitoring [14].

Simultaneous identification of proteomic signatures could provide novel biomarker panels for diagnostic and personalized treatment of different types of brain tumors, including glioblastoma. Personalized medicine is starting to gain importance in clinical care, already having recorded a series of achievements in several types of cancer; nonetheless, in brain tumors it is still at an early stage [15].

The current clinical diagnostic of glioma is based mainly on imaging diagnostic and anatomopathological findings in biopsy pieces [16], including markers such as GFAP or Ki-67 to indicate proliferative activity, which, for this type of cerebral tumor, is especially high. Additionally, genetic markers, such as loss of heterozygosity on chromosome 10q and mutations in EGFR signaling – and Akt – signaling coding genes can be identified [17]. Although present, some of them with high incidence, these markers are not useful for diagnostic, as they can be present in other types of cancer (e.g. EGFR mutations in non-small cell lung cancer) and, furthermore, therapeutic attempts with monoclonal antibodies targeting altered signaling pathways (EGFR singaling, VEGF singaling) failed to improve clinical outcome [15]. The epigenetic mark of O^6^-methylguanine–DNA methyltransferase (MGMT) promoter methylation is also taken under consideration for glioblastoma patients, as it was proven to correlate with survival, following temolozomide treatment [18]. Most newly identified markers for glioblastoma, such as mutations in IDH [19] or TET genes [20] are also used mostly for prognostic purposes and putative identification of therapeutic targets.

So far, there are several proteomic and biochemical multi-step technologies used for discovery and validation of biomarkers, such as SELDI-ToF MS, LC-MS/MS, 1D PAGE, and Western blot. For the current research, SELDI-ToF MS was preferred because it is an effective array technology, allowing complex analysis of biological materials and providing a rapid protein expression profile useful for biomarker identification [21,22]. Furthermore, SELDI-ToF MS technology has been successfully applied in biomarker discovery for a number of cancer types, such as acute myeloid leukemia [23,24], pancreatic cancer [25], lung cancer [26,27], ovarian cancer [28] and gastric adenocarcinoma [29], but insufficiently reported in glioblastoma [30].

A current desiderate in glioblastoma diagnostic is generation of a serum panel of markers, that would expedite the diagnostic process and bypass the mandatory procurement of bioptic material. Not GFAP, but GFAP autoantibodies were very recently demonstrated to be a useful serum marker for glioma patients [3], possibly due to the fact that blood brain barrier (otherwise impermeable for antibodies) is altered in advanced or aggressive disease.

In the present study, SELDI-ToF MS technology was performed in combination with LC-MS/MS to analyze and compare the serum samples of glioblastoma patients in order to discover novel biomarker panels.

As previously reported by other groups, serum of glioblastoma patients contain specific peptide peaks that can be selected to differentiate between glioblastoma and other types of cerebral tumors [31]. Out of all identified peaks, we selected those with the highest sensitivity and further identified them through LC/MC as proteins S100A8, S100A9 and CXCL4. Interestingly, although these proteins have been previously detected and reported in other cancers, as further discussed, they are not exclusively cancer-related markers, but classically described as inflammation-related factors. This is but one of the consequences of accelerated knowledge accumulation of the last years, during which new and exciting connections between cellular mechanisms have been revealed.

Our results revealed 3 candidate biomarkers useful in glioblastoma diagnostic: CXCL4, S100A8 and S100A9, with increased serum levels/tissue overexpression in glioblastoma *versus* control. All of these molecules have been previously identified as possible biomarkers in different tumor types [32].

For the first time, increased levels of CXCL4/PF-4 were found in coronary artery disease, being used as a measure of platelet activation. In patients with bone metastasis, including patients with breast and prostate cancer, elevated plasma PF-4 levels have been identified, being positively correlated with increased TGF-β levels [33].

Also, CXCL4/PF-4 has been proposed as a biomarker of early tumor growth in different tumors types, and appears to be up-regulated in human liposarcoma, mammary adenocarcinoma, osteosarcoma [7,34] and in other hematologic disorders [35]. The up-regulation of CXCL4/PF-4 in these tumor types may be a way to counterbalance angiogenic growth factors [36]. Pinedo et al. have suggested that platelets contribute to tumor-induced angiogenesis and that interactions between platelets and endothelium play an important role in tumor growth [37]. Indeed, a reduced blood flow was observed in tumors, presumably caused by raised interstitial pressure and hyperpermeability of tumor capillaries in response to platelet-derived VEGF.

CXCL4/PF4 was identified and confirmed as a new discriminating marker for pancreatic cancer using MALDI-TOF MS. In combination with the conventional markers (CA 19–9 and CEA), CXCL4 improves the diagnostic power of tumor biomarker testing [38]. In 2010, Poruk *et al.* showed that in pancreatic adenocarcinoma, serum PF-4 could be considered a prognostic factor for survival and increased risk for the development of venous thromboembolism [39].

Recent studies suggest that CXCL4 is overexpressed in alcoholic liver disease, human liver fibrosis, and also elevated in patients with viral hepatitis compared to healthy individuals [32,40]. There are also many studies which argue whether CXCL4/PF-4 is involved in angiogenesis and carcinogenesis [32].

In 2012, Peterson *et al.* showed that patients with colorectal cancer present higher levels of angiogenesis factors PF-4, PDGF and VEGF, which could be used for a possible early diagnosis of this type of cancer [41].

There are also reports indicating that CXCL4 was decreased in samples collected from cancer patients. CXCL4/PF-4 was found to be significantly decreased in patients suffering from pancreatic cancer [38], in sera of metastatic prostate cancer patients compared to healthy persons or non-metastatic prostate cancer patients [42] and in metastatic cancer patients (colorectal cancer, renal cell cancer, malignant fibrous histiocytoma, leiomyosarcoma, peripheral neuroectodermal tumor) compared to controls [43]. To identify a stage-specific marker, mass spectrometry based mass profiling was combined with a whole-protein based top-down separation strategy also combined with multivariate analysis. A single protein - CXCL4 was found to be significantly decreased, therefore chosen as the primary candidate for further analysis [42]. In prostate cancer cell lines, CXCL4/PF-4 and CXCL10/IP10, both ligands for CXCR3 receptor promote cell motility and invasiveness [44].

Increased levels of S100A8 and S100A9 were found in many pathological conditions associated with inflammation; therefore, they may have a possible role in tumorigenesis [45]. It is considered that S100A8 and S100A9 can exert opposing roles in inflammation, their expression being induced by VEGF-A, TGFβ, TNFα and also by anti-inflammatory mediator IL-10 [46]. Increasing evidence suggests that changes in the expression and/or function of S100 proteins may be critical during cancer development [26].

S100A8/A9 are strongly up-regulated in breast [47], lung [48], gastric [49], colorectal [50], pancreatic [51], skin cancers [52] and prostate cancer [53], in inflammation associated with cancer [45] and altered S100A9 expression in carcinomas can lead to chemoresistance [54]. Tumor cells produce S100A8/A9 in response to stimuli [55-57]. For example, phorbol esters stimulate secretion of S100A8/A9 by prostate cancer cells [57] and S100A9 expression is induced in hepatocellular carcinoma through activation of NF-KB signaling [56]. In addition, S100A8/A9 could also be released by tumor cell necrosis following hypoxia within growing tumors. Regardless of the source, it now appears that they play important roles in both inflammation-induced cancer and cancers-induced inflammation, and mediate concentration-dependent anti- or pro-tumor responses.

S100A8 and S100A9 act as danger associated molecular pattern (DAMP) molecules modulating host immune responses and promoting tumorigenesis and progression to malignancy [45,58].

Nemeth *et al*., 2009 detected increased co-expression of S100A8 and S100A9 proteins in human hepatocellular carcinoma tissue, and in the hepatocellular carcinoma cell line Hep3B. S100A8 and S100A9 are NF-kB target genes in human HCC cells during inflammation-associated liver carcinogenesis and increased co-expression of both proteins supports malignant progression by activation of ROS-dependent signaling pathways and protection from cell death [56].

Some studies demonstrate that extracellular S100A8/A9 complex exhibits growth-inhibitory properties and promotes cytotoxicity and apoptosis in many different human tumor cells, strongly indicating that S100A8/A9 elicit powerful anti-tumor responses, and that the cell death pathway mediated by these proteins might therefore provide targets for developing novel therapeutic tools against cancers. Recent *in vitro* and *in vivo* studies indicate that S100A8/A9 mediate several pro-tumor responses; the two apparently opposite effects may be dependent on its extracellular concentration and activation of different signaling pathways [46].

Decreased expressions of S100A9 and S100A8 were observed in human cervical squamous cell carcinoma. In CaSki human cervical cancer cells S100A8/A9 treatment induces apoptosis and inhibits migration of CaSki cells; S100A8/A9 also reduced the expression of matrix metalloproteinase (MMP)-2 in CaSki cells [59].

S100A9 expression was correlated with an early stage cancer and a better prognosis in patients with gastric cancer [60]. There are studies supporting those S100A proteins can induce sensitivity/resistance to chemotherapy in cancer [61]. In human gastric cancer cell line SNU484, S100A8 and S100A9 inhibition was linked with decreased of invasive and migratory phenotypes of tumoral cells. S100A8 and S100A9 are also involved in transcriptional activation of MMP-2 gene [62-64]. Another *in vitro* study conducted on gastric cancer cell line underline that S100A8 and S100A9 expression are associated with a decrease in lymph node metastasis and these proteins can be used as biomarkers in gastric adenocarcinoma [64]. Based on differential expression and subcellular localization of S100A9 and S100A8/A9 some studies suggest that only S100A9 plays a role in gastric carcinogenesis [60].

In breast cancer, S100A8 and S100A9 are also linked with tumor progression being involved in regulating cancer cell behavior through extracellular and intracellular signaling pathways. One study conducted on breast cancer cell line showed that extracellular treatments with S100A8 and S100A9 proteins induce cell proliferation, while intracellular recombinant expression of S100A8 and S100A9 block cancer cells grow. Moreover, S100A8 and S100A9 seem to suppress breast cancer by activating mesenchymal to epithelial transition [65].

In 2012 Li C. et al. showed that S100A8 and S100A9 proteins at relatively low concentrations can promote angiogenesis by increasing proliferation, migration, and tube formation of vascular endothelial cells in human umbilical vascular endothelial cell line [66]. *In vitro* study showed that S100A8/A9 expression results in infiltration of immune cells, especially neutrophils in tumors of the mouse injected with lung cancer cells [67].

Less is known about the expression of those candidate biomarkers in glioblastoma. One study, performed by Gautam *et al.*, using plasma samples of glioblastoma patients, using an iTRAQ based LC-MS/MS approach, has observed significantly elevated levels of two representative proteins, ferritin light chain (FTL) and S100A9. These proteins are useful as starting point for further clinical investigations for the development of plasma-based biomarker panels for glioblastoma [30].

A better understanding of molecular pathways mediated by these biomarkers will provide exciting opportunities for the study and design of novel cancer therapy.

## Conclusions

In summary, we have identified a novel panel of protein biomarkers that could discriminate glioblastoma patients from control. The above mentioned biomarkers obtained with SELDI-ToF MS and further identified by LC-MS/MS were validated by ELISA and Western blot.

We have shown that SELDI-ToF MS can be employed to explore the proteome of a complex disease, like glioblastoma and have obtained protein profiles of differentialy expressed proteins.

In this study, a panel of three proteins: S100A8, S100A9 and CXCL4 was selected and then examined. Overexpression of these proteins and their presence in patients with glioblastoma compared to the control group were confirmed by ELISA. The study has continued with validation of the above mentioned molecules by Western blot, where proteins S100-A9 and CXCL4 were found in significantly higher amounts in tumor samples compared to the peritumoral tissue.

While it is unlikely for a single biomarker to be highly effective for detecting cancer pathology and survival outcome for patients, our data demonstrated an alternative and efficient approach to predict cancer progression and survival outcome of the glioblastoma patients using a novel combination of multiple biomarkers.

The use of proteomic technology may provide a completely novel tool for early diagnosis improvement, targeted therapy and relapse prediction in glioblastoma patients.

## Materials and methods

### Samples collection and lot design

A total of 35 patients (14 females and 21 males) with anatomopathological confirmed diagnostic of glioblastoma multiforme stage IV (GBM) were included in the study, along with 30 healthy controls. Patients underwent surgery at National Institute of Neurological and Neurovascular Diseases, Bucharest, Romania and ELIAS Emergency Neurosurgery Department, Bucharest, Romania while healthy controls serum samples were collected at Diagnosis Center of "Victor Babeş" National Institute of Pathology, Bucharest, Romania. Tumoral and peritumoral tissues were collected in sterile saline solution and stored at −80°C. Total peripheral blood was collected in vacutainer tubes without anticoagulant (Systems, Becton Dickinson). Serum samples were aliquoted and stored at −80°C until analysis. Written informed consent has been obtained upon sample collection according to Helsinki II Declaration and the study has been approved by the local ethics committee.

### SELDI-ToF MS - optimization of parameters

All serum samples were treated with denaturation buffer consisting of 9.5 M urea, 2% (w/v) CHAPS and 1% (w/v) DTT and stored at −80°C until analysis by SELDI-ToF MS.

SELDI-ToF MS was carried out using binding and wash buffers of varying pH and ionic strength to compare the impact of increased or decreased stringency of protein binding. Two different arrays in combination with different buffers and pool sera from glioblastoma patients and controls were evaluated.

Essentially, samples were diluted in loading buffers over a pH range (at 0.5 pH intervals) and applied to spots on strong anionic exchange (Q10) and weak cationic exchange (CM10) arrays. For CM10 arrays the pH range was 3.5-7.0 (50 mM ammonium acetate for pH 3.5-5.5 and 50 mM sodium phosphates for pH 6.0-7.0). pH range for Q10 arrays were between 4.5-8.0 (50 mM ammonium acetate for pH 5.0-5.5; 50 mM sodium phosphates for pH 6.0-7.5 and 50 mM Tris buffer for pH 8.0-8.5). Sinapinic acid energy absorbing matrix was precipitated onto sample spots and allowed to air dry prior to mass spectrometry.

Serum was first diluted 1/10 in denaturation buffer, and after short incubation on ice, samples were diluted 1/10 in binding buffer. Prior to sample loading CM10 and Q10 arrays were equilibrated with 200 μL binding solution for 10 minutes. An amount of 100 μL diluted serum samples was added to array and then incubated with vigorous shaking for 1 hour at room temperature. After incubation the arrays have been washed 3 times with binding buffer and one time with de-ionized water (5 minutes shaking). Finally, 0.5 μL sinapinic acid solution was added twice to each spot and allowed to air-dry.

### SELDI-ToF MS - analysis

Arrays were read on a Proteinchip Enterprise 4000 system, BioRad (Hercules, CA, USA). The following settings were followed in both SELDI readers: target m/z 5 kDa, matrix attenuation at 2.5 kDa and mass range between 0–100 kDa. External calibration was performed using protein standards comprised of recombinant hirudin (6.96 kDa), equine cytochrome (12.23 kDa), equine myoglobin (16.95 kDa), and carbonic anhydrase (29.00 kDa).

Mass accuracy (m/Δm) was calculated at ≤ 0.02% throughout the entire experimental mass range. Noise definitions were adjusted to eliminate chemical noise in the low mass range, the area below the detector blinding setting (m/z 2,500) was excluded. Only peaks with a signal-to-noise (S/N) ratio of ≥ 5 and a valley depth ≥ 3 were considered for clustering. Qualified peaks which were present in ≥ 10% of the spectra were used to generate peak clusters. Unlabeled spectra were then labelled at the average mass of the cluster so that a peak intensity value was obtained for each spectrum. The mass window for each cluster was set at 0.3% of the peak mass for spectra optimized for low mass (0–30 kDa) and at 2% of the peak mass for spectra optimized for high mass (30–100 kDa). Qualified mass peaks (S/N > 5) within m/z range of 2.5-100 kDa were auto detected. Peak clustering was completed using a second-pass peak selection (S/N > 2, within 0.3% mass window) and estimated peaks added.

### Biomarker delineation

All individual serum samples were analysed at the two selected pHs (4.5 and 6.0 for CM10). Spectral data were analysed using ProteinChip Data Manager v. 3.0.7 Software to generate peak mass clusters and the delineation of candidate biomarker peaks. ANOVA Tests were performed to identify significant differences between data derived from different groups. Biomarkers were accepted as candidate if p < 0.05. The manual inspection of spectra containing candidate biomarkers was employed in order to determine if they had the characteristics of proteins liable to give reliable identification following separation on 1D gels and analysis by tryptic digestion and LC-MS/MS. Such peaks should preferably be more than 5 kDa and have the peak intensity more than 5 times the background.

### Sample fractionation

Due to the high complexity of serum, the large range of abundance and the dominance by just few proteins including albumin and IgG downstream purification of candidate biomarkers was performed following sample pooling and fractionation using buffer conditions commensurate with those used on SELDI arrays to delineate those biomarkers. Pools of samples were constructed from contributions of those samples which had the highest relative levels of each biomarker. These pools were loaded onto ProteinChip Q filtration plate (Bio-Rad) and discontinuous fractions eluted by increasing salt strength buffers and collected for analysis. After buffer exchange small aliquots of each fraction were applied to SELDI CM10 arrays at pH and salt strength conditions commensurate with the identification of original SELDI biomarkers.

### 1D PAGE

Fractionated samples were concentrated and buffer exchanged in 50 mM Tris pH 8.0 in 3 kDa cut-off filters down to ~50 μL. The samples were further concentrated by reducing the volume down to ~20 μL in a speed vacuum; 10 μL of sample was made up to 20 μL with 8 μL of 4x sample buffer (Invitrogen) and 2 μL 500 mM DTT. The sample was boiled for 10 minutes at 70°C. The Novex gel electrophoresis system (Invitrogen, Carlsbad, CA, USA) was used to run the gels with a 12% BisTris pre-cast gel and MES running buffer. SeeBlue plus2 pre-stained molecular weight markers (Invitrogen) were run on each gel. Gels were fixed and stained in Colloidal Coomassie Blue. Concentrated samples were loaded on gels without balancing total protein across fractions. This was because each fraction contained different amounts of total protein.

### Protein digestion

Proteins bands were digested (non-automated) with trypsin. Specifically gel bands were washed with 100 mM Ammonium Bicarbonate (Ambic) followed by acetonitrile (ACN). Protein bands were then reduced and alkylated with 10 mM DTT and 55 mM IAA respectively, both dissolved in 100 mM Ambic. Gel bands were destained with 50% 100 mM Ambic/50% ACN before a final wash cycle of 100 mM Ambic and ACN. Samples were lyophilised to dryness in a centrifugal evaporator and rehydrated in trypsin solution (Promega sequencing grade; 20 μg aliquot was resuspended to 100 ng/μL with 0.1% TFA, immediately prior to use, this was diluted to 13 ng/μl with 50 mM Ambic). Samples were incubated at 4°C for 20 minutes, unabsorbed trypsin solution was removed and the gel pieces were immersed in a minimal volume of 50 mM Ambic. Samples were left to digest at 37°C for 2 hours followed by overnight incubation at room temperature. Supernatant containing peptides was decanted into a new tube and the gel pieces washed with two cycles of 100 mM Ambic and ACN, each time pooling the extraction solution with the initial supernatant. The pooled supernatant was lyophilised to dryness and resuspended in 25 μL of 5% ACN/0.1% formic acid for MS analysis.

### LC-MS/MS analysis

Peptides were analysed by LC-MS/MS using a Surveyor LC system and LCQ Deca XP Plus (Thermo Scientific). Briefly, peptides were resolved by reverse phase chromatography (Biobasic column, Thermo Scientific; 180 uM × 15 mm) over a 30 min ACN gradient at a flow rate of 3 μL/min. Peptides were ionised by electrospray ionisation and MS/MS was acquired on ions dependant on their charge state and intensity. Quality control checks for the optimal performance of the instrumentation were in place. Mass accuracy and sensitivity of the MS was confirmed with the direct infusion of glufibriopeptide (2.5 pmoles/μL) and LC-MS/MS performance was assessed with a digest of BSA. Sensitivity, retention time, peptides identified and protein sequence coverage were all within the specified ranges. BSA quality control checks were performed prior to the analysis of the sample and post-acquisition.

### Validation by ELISA assays

Validation of the selected biomarkers were conducted with ELISA. The concentration of the proteins in the 35 glioblastoma serum samples were quantified using the commercial ELISA kits (Human S100 Calcium Binding Protein A8/Calgranulin A: Wuhan Eiaab Science, China; Human S100 Calcium Binding Protein A9/Calgranulin B: Wuhan Eiaab Science, China; Human PF-4: RayBiotech, USA) according to the manufacturer’s instructions. For each biomarker, the samples were assayed in triplicate and the average concentrations were used for statistical analysis.

### Validation by Western blot assays

Tumoral-derived and peritumoral tissues from patients were lysed on ice, in a buffer containing 50 mM Tris–HCl pH 7.4, 150 mM NaCl, 1% Triton X-100, 0.5% sodium deoxycholate, 0.1% SDS, 2 mM EDTA and 1% protease inhibitors cocktail (Sigma-Aldrich); tissues lysates:buffer at a ratio 1:2 (w/v). The protein concentration was determined using the DC protein assay reagent (Bio-Rad, Hercules, CA, USA) and 25 μg of protein was loaded on a 12% SDS-PAGE gel. Electrophoresis was run at 20 mA/gel and separated proteins were subsequently blotted on nitrocellulose membranes at 100 V for 1 h, on ice. After 1 hour blocking with BSA 2.5% in TBS, membranes were incubated overnight with primary antibodies PF-4 Antibody (V-15): sc-23519; S100-A9/Calgranulin B (H-90): sc-20173 (Santa Cruz Biotechnology, CA), diluted 1:200 in washing buffer (10 mM Tris–HCl, pH 8.0, 150 mM NaCl, 0.1% Tween 20) supplemented with 2% BSA-TTBS. Following incubation with primary antibody, blots were washed and incubated for 1 h with suitable secondary antibody: HRP-conjugated goat anti-rabbit for Calgranulin B (1:10.000 dilution) and HRP-conjugated donkey anti-goat for PF-4 (1:5000 dilution) (Santa Cruz Biotechnology, CA). Membranes were incubated for 5 minute in ECL reagents (Pierce, Rockford, USA) and exposed to film.

## References

[1] Kocevar N, Hudler P, Komel R (2013). The progress of proteomic approaches in searching for cancer biomarkers. N Biotechnol.

[2] Sathornsumetee S, Reardon DA, Desjardins A, Quinn JA, Vredenburgh JJ, Rich JN (2007). Molecularly targeted therapy for malignant glioma. Cancer.

[3] Wei P, Zhang W, Yang LS, Zhang HS, Xu XE, Jiang YH, Huang FP, Shi Q (2013). Serum GFAP autoantibody as an ELISA-detectable glioma marker. Tumour Biol.

[4] Van Meir EG, Hadjipanayis CG, Norden AD, Shu HK, Wen PY, Olson JJ (2010). Exciting new advances in neuro-oncology: the avenue to a cure for malignant glioma. CA Cancer J Clin.

[5] Brat DJ, Castellano-Sanchez A, Kaur B, Van Meir EG (2002). Genetic and biologic progression in astrocytomas and their relation to angiogenic dysregulation. Adv Anat Pathol.

[6] Kalinina J, Peng J, Ritchie JC, Van Meir EG (2011). Proteomics of gliomas: initial biomarker discovery and evolution of technology. Neuro Oncol.

[7] Cervi D, Yip TT, Bhattacharya N, Podust VN, Peterson J, Abou-Slaybi A, Naumov GN, Bender E, Almog N, Italiano JE, Folkman J, Klement GL (2008). Platelet-associated PF-4 as a biomarker of early tumor growth. Blood.

[8] Chen S, Zhao H, Deng J, Liao P, Xu Z, Cheng Y (2013). Comparative proteomics of glioma stem cells and differentiated tumor cells identifies S100A9 as a potential therapeutic target. J Cell Biochem.

[9] Engwegen JY, Gast MC, Schellens JH, Beijnen JH (2006). Clinical proteomics: searching for better tumour markers with SELDI-TOF mass spectrometry. Trends Pharmacol Sci.

[10] Ehmann M, Felix K, Hartmann D, Schnolzer M, Nees M, Vorderwulbecke S, Bogumil R, Buchler MW, Friess H (2007). Identification of potential markers for the detection of pancreatic cancer through comparative serum protein expression profiling. Pancreas.

[11] Albulescu R, Codrici E, Popescu ID, Mihai S, Necula LG, Petrescu D, Teodoru M, Tanase CP: **Cytokine Patterns in Brain Tumour Progression.** *Mediators Inflamm* 2013, ID 979748, doi:10.1155/2013/979748. 10.1155/2013/979748 PMC3707225 23864770

[12] Jaunalksne I, Donina S, Pirtnieks A, Grusina-Ujumaza J, Spaka I, Babjoniseva A, Krievins D (2012). Dynamics of CXC group chemokine platelet factor 4 (PF4) plasma levels in non-small cell lung cancer (NSCLC). J Transl Med.

[13] Pilatova K, Greplova K, Demlova R, Bencsikova B, Klement GL, Zdrazilova-Dubska L (2013). Role of platelet chemokines, PF-4 and CTAP-III, in cancer biology. J Hematol Oncol.

[14] Somasundaram K, Nijaguna MB, Kumar DM (2009). Serum proteomics of glioma: methods and applications. Expert Rev Mol Diagn.

[15] Tanase CP, Enciu AM, Mihai S, Neagu AI, Calenic B, Cruceru ML (2013). Anti-cancer Therapies in High Grade Gliomas. Curr Proteonomics.

[16] Ahmed R, Oborski MJ, Hwang M, Lieberman FS, Mountz JM (2014). Malignant gliomas: current perspectives in diagnosis, treatment, and early response assessment using advanced quantitative imaging methods. Canc Manag Res.

[17] Louis DN, Ohgaki H, Wiestler OD, Cavenee WK, Burger PC, Jouvet A, Scheithauer BW, Kleihues P (2007). The 2007 WHO classification of tumours of the central nervous system. Acta Neuropathol.

[18] Donson AM, Addo-Yobo SO, Handler MH, Gore L, Foreman NK (2007). MGMT promoter methylation correlates with survival benefit and sensitivity to temozolomide in pediatric glioblastoma. Pediatr Blood Cancer.

[19] van den Bent MJ, Dubbink HJ, Marie Y, Brandes AA, Taphoorn MJ, Wesseling P, Frenay M, Tijssen CC, Lacombe D, Idbaih A, van Marion R, Kros JM, Dinjens WN, Gorlia T, Sanson M (2010). IDH1 and IDH2 mutations are prognostic but not predictive for outcome in anaplastic oligodendroglial tumors: a report of the European Organization for Research and Treatment of Cancer Brain Tumor Group. Clin Cancer Res.

[20] Orr BA, Haffner MC, Nelson WG, Yegnasubramanian S, Eberhart CG (2012). Decreased 5-hydroxymethylcytosine is associated with neural progenitor phenotype in normal brain and shorter survival in malignant glioma. PLoS One.

[21] Popescu ID, Albulescu R, Raducan E, Dinischiotu A, Tanase C (2010). Applications of SELDI-TOF technology in cancer biomarkers discovery. Romanian Biotechnol Lett.

[22] Azzam S, Broadwater L, Li S, Freeman EJ, McDonough J, Gregory RB (2013). A SELDI mass spectrometry study of experimental autoimmune encephalomyelitis: sample preparation, reproducibility, and differential protein expression patterns. Proteome Sci.

[23] Forshed J, Pernemalm M, Tan CS, Lindberg M, Kanter L, Pawitan Y, Lewensohn R, Stenke L, Lehtio J (2008). Proteomic data analysis workflow for discovery of candidate biomarker peaks predictive of clinical outcome for patients with acute myeloid leukemia. J Proteome Res.

[24] Bai J, He A, Zhang W, Huang C, Yang J, Yang Y, Wang J, Zhang Y (2013). Potential biomarkers for adult acute myeloid leukemia minimal residual disease assessment searched by serum peptidome profiling. Proteome Sci.

[25] Navaglia F, Fogar P, Basso D, Greco E, Padoan A, Tonidandel L, Fadi E, Zambon CF, Bozzato D, Moz S, Seraglia R, Pedrazzoli S, Plebani M (2009). Pancreatic cancer biomarkers discovery by surface-enhanced laser desorption and ionization time-of-flight mass spectrometry. Clin Chem Lab Med.

[26] Han KQ, Huang G, Gao CF, Wang XL, Ma B, Sun LQ, Wei ZJ (2008). Identification of lung cancer patients by serum protein profiling using surface-enhanced laser desorption/ionization time-of-flight mass spectrometry. Am J Clin Oncol.

[27] Lin Q, Peng Q, Yao F, Pan XF, Xiong LW, Wang Y, Geng JF, Feng JX, Han BH, Bao GL, Yang Y, Wang X, Jin L, Guo W, Wang JC (2012). A classification method based on principal components of SELDI spectra to diagnose of lung adenocarcinoma. PLoS One.

[28] Wegdam W, Moerland PD, Meijer D, de Jong SM, Hoefsloot HC, Kenter GG, Buist MR, Aerts JM (2012). A critical assessment of SELDI-TOF-MS for biomarker discovery in serum and tissue of patients with an ovarian mass. Proteome Sci.

[29] Liu C, Pan C, Liang Y (2012). Screening and identification of serum proteomic biomarkers for gastric adenocarcinoma. Exp Ther Med.

[30] Gautam P, Nair SC, Gupta MK, Sharma R, Polisetty RV, Uppin MS, Sundaram C, Puligopu AK, Ankathi P, Purohit AK, Chandak GR, Harsha HC, Sirdeshmukh R (2012). Proteins with altered levels in plasma from glioblastoma patients as revealed by iTRAQ-based quantitative proteomic analysis. PLoS One.

[31] Li Z, Lu H, Yang J, Zeng X, Zhao L, Li H, Liao Q, Peng S, Zhou M, Wu M, Xiang J, Wang Y, Li G (2013). Analysis of the raw serum peptidomic pattern in glioma patients. Clin Chim Acta.

[32] Sandset PM (2012). CXCL4-platelet factor 4, heparin-induced thrombocytopenia and cancer. Thromb Res.

[33] Baselga J, Rothenberg ML, Tabernero J, Seoane J, Daly T, Cleverly A, Berry B, Rhoades SK, Ray CA, Fill J, Farrington DL, Wallace LA, Yingling JM, Lahn M, Arteaga C, Carducci M (2008). TGF-beta signalling-related markers in cancer patients with bone metastasis. Biomarkers.

[34] Vandercappellen J, Van Damme J, Struyf S (2011). The role of the CXC chemokines platelet factor-4 (CXCL4/PF-4) and its variant (CXCL4L1/PF-4var) in inflammation, angiogenesis and cancer. Cytokine Growth Factor Rev.

[35] Kowalska MA, Rauova L, Poncz M (2010). Role of the platelet chemokine platelet factor 4 (PF4) in hemostasis and thrombosis. Thromb Res.

[36] Van Raemdonck K, Gouwy M, Lepers SA, Van Damme J, Struyf S (2014). CXCL4L1 and CXCL4 signaling in human lymphatic and microvascular endothelial cells and activated lymphocytes: involvement of mitogen-activated protein (MAP) kinases, Src and p70S6 kinase. Angiogenesis.

[37] Pinedo HM, Verheul HM, D'Amato RJ, Folkman J (1998). Involvement of platelets in tumour angiogenesis?. Lancet.

[38] Fiedler GM, Leichtle AB, Kase J, Baumann S, Ceglarek U, Felix K, Conrad T, Witzigmann H, Weimann A, Schutte C, Hauss J, Buchler M, Thiery J (2009). Serum peptidome profiling revealed platelet factor 4 as a potential discriminating Peptide associated with pancreatic cancer. Clin Cancer Res.

[39] Poruk KE, Firpo MA, Huerter LM, Scaife CL, Emerson LL, Boucher KM, Jones KA, Mulvihill SJ (2010). Serum platelet factor 4 is an independent predictor of survival and venous thromboembolism in patients with pancreatic adenocarcinoma. Cancer Epidemiol Biomarkers Prev.

[40] Zaldivar MM, Pauels K, von Hundelshausen P, Berres ML, Schmitz P, Bornemann J, Kowalska MA, Gassler N, Streetz KL, Weiskirchen R, Trautwein C, Weber C, Wasmuth HE (2010). CXC chemokine ligand 4 (Cxcl4) is a platelet-derived mediator of experimental liver fibrosis. Hepatology.

[41] Peterson JE, Zurakowski D, Italiano JE, Michel LV, Connors S, Oenick M, D'Amato RJ, Klement GL, Folkman J (2012). VEGF, PF4 and PDGF are elevated in platelets of colorectal cancer patients. Angiogenesis.

[42] Lam YW, Mobley JA, Evans JE, Carmody JF, Ho SM (2005). Mass profiling-directed isolation and identification of a stage-specific serologic protein biomarker of advanced prostate cancer. Proteomics.

[43] Wiesner T, Bugl S, Mayer F, Hartmann JT, Kopp HG (2010). Differential changes in platelet VEGF, Tsp, CXCL12, and CXCL4 in patients with metastatic cancer. Clin Exp Metastasis.

[44] Wu Q, Dhir R, Wells A (2012). Altered CXCR3 isoform expression regulates prostate cancer cell migration and invasion. Mol Cancer.

[45] Gebhardt C, Nemeth J, Angel P, Hess J (2006). S100A8 and S100A9 in inflammation and cancer. Biochem Pharmacol.

[46] Srikrishna G (2012). S100A8 and S100A9: new insights into their roles in malignancy. J Innate Immun.

[47] Parris TZ, Kovacs A, Aziz L, Hajizadeh S, Nemes S, Semaan M, Forssell-Aronsson E, Karlsson P, Helou K (2014). Additive effect of the AZGP1, PIP, S100A8 and UBE2C molecular biomarkers improves outcome prediction in breast carcinoma. Int J Cancer.

[48] Xu Y, Cao LQ, Jin LY, Chen ZC, Zeng GQ, Tang CE, Li GQ, Duan CJ, Peng F, Xiao ZQ, Li C (2012). Quantitative proteomic study of human lung squamous carcinoma and normal bronchial epithelial acquired by laser capture microdissection. J Biomed Biotechnol.

[49] Hu Y, Fan B, Zhang LH, Cheng XJ, Niu ZJ, Ji JF (2013). Clinical significance of S100A8 and S100A9 expression in gastric cancer. Zhonghua Yi Xue Za Zhi.

[50] Duan L, Wu R, Ye L, Wang H, Yang X, Zhang Y, Chen X, Zuo G, Weng Y, Luo J, Tang M, Shi Q, He T, Zhou L (2013). S100A8 and S100A9 are associated with colorectal carcinoma progression and contribute to colorectal carcinoma cell survival and migration via Wnt/beta-catenin pathway. PLoS One.

[51] Chen KT, Kim PD, Jones KA, Devarajan K, Patel BB, Hoffman JP, Ehya H, Huang M, Watson JC, Tokar JL, Yeung AT (2014). Potential prognostic biomarkers of pancreatic cancer. Pancreas.

[52] McNeill E, Hogg N (2014). S100A9 has a protective role in inflammation-induced skin carcinogenesis. Int J Cancer.

[53] Grebhardt S, Veltkamp C, Strobel P, Mayer D (2012). Hypoxia and HIF-1 increase S100A8 and S100A9 expression in prostate cancer. Int J Cancer.

[54] Ju W, Yoo BC, Kim IJ, Kim JW, Kim SC, Lee HP (2009). Identification of genes with differential expression in chemoresistant epithelial ovarian cancer using high-density oligonucleotide microarrays. Oncol Res.

[55] Sinha P, Okoro C, Foell D, Freeze HH, Ostrand-Rosenberg S, Srikrishna G (2008). Proinflammatory S100 proteins regulate the accumulation of myeloid-derived suppressor cells. J Immunol.

[56] Nemeth J, Stein I, Haag D, Riehl A, Longerich T, Horwitz E, Breuhahn K, Gebhardt C, Schirmacher P, Hahn M, Ben-Neriah Y, Pikarsky E, Angel P, Hess J (2009). S100A8 and S100A9 are novel nuclear factor kappa B target genes during malignant progression of murine and human liver carcinogenesis. Hepatology.

[57] Hermani A, De Servi B, Medunjanin S, Tessier PA, Mayer D (2006). S100A8 and S100A9 activate MAP kinase and NF-kappaB signaling pathways and trigger translocation of RAGE in human prostate cancer cells. Exp Cell Res.

[58] Salama I, Malone PS, Mihaimeed F, Jones JL (2008). A review of the S100 proteins in cancer. Eur J Surg Oncol.

[59] Qin F, Song Y, Li Z, Zhao L, Zhang Y, Geng L (2010). S100A8/A9 induces apoptosis and inhibits metastasis of CasKi human cervical cancer cells. Pathol Oncol Res.

[60] Fan B, Zhang LH, Jia YN, Zhong XY, Liu YQ, Cheng XJ, Wang XH, Xing XF, Hu Y, Li YA, Du H, Zhao W, Niu ZJ, Lu AP, Li JY, Ji JF (2012). Presence of S100A9-positive inflammatory cells in cancer tissues correlates with an early stage cancer and a better prognosis in patients with gastric cancer. BMC Cancer.

[61] Jin L, Shen Q, Ding S, Jiang W, Jiang L, Zhu X (2012). Immunohistochemical expression of Annexin A2 and S100A proteins in patients with bulky stage IB-IIA cervical cancer treated with neoadjuvant chemotherapy. Gynecol Oncol.

[62] Yong HY, Moon A (2007). Roles of calcium-binding proteins, S100A8 and S100A9, in invasive phenotype of human gastric cancer cells. Arch Pharm Res.

[63] Ghavami S, Chitayat S, Hashemi M, Eshraghi M, Chazin WJ, Halayko AJ, Kerkhoff C (2009). S100A8/A9: a Janus-faced molecule in cancer therapy and tumorgenesis. Eur J Pharmacol.

[64] Choi JH, Shin NR, Moon HJ, Kwon CH, Kim GH, Song GA, Jeon TY, Kim DH, Park do Y (2012). Identification of S100A8 and S100A9 as negative regulators for lymph node metastasis of gastric adenocarcinoma. Histol Histopathol.

[65] Cormier K, Harquail J, Ouellette RJ, Tessier PA, Guerrette R, Robichaud GA (2013). Intracellular expression of inflammatory proteins S100A8 and S100A9 leads to epithelial-mesenchymal transition and attenuated aggressivity of breast cancer cells. Anticancer Agents Med Chem.

[66] Li C, Li S, Jia C, Yang L, Song Z, Wang Y (2012). Low concentration of S100A8/9 promotes angiogenesis-related activity of vascular endothelial cells: bridges among inflammation, angiogenesis, and tumorigenesis?. Mediators Inflamm.

[67] Grebhardt S, Muller-Decker K, Bestvater F, Hershfinkel M, Mayer D (2013). Impact of S100A8/A9 expression on prostate cancer progression in vitro and in vivo. J Cell Physiol.

